# Weight Loss at First Month and Development of Tolerance as Possible Predictors of 30 mg Phentermine Efficacy at 6 Months

**DOI:** 10.3390/jpm11121354

**Published:** 2021-12-12

**Authors:** Héctor Isaac Rocha-González, Lidia Elizabeth De la Cruz-Álvarez, Ashuin Kammar-García, Samuel Canizales-Quinteros, Juan Carlos Huerta-Cruz, Lina Marcela Barranco-Garduño, Juan Gerardo Reyes-García

**Affiliations:** 1 Sección de Estudios de Posgrado e Investigación, Escuela Superior de Medicina, Instituto Politécnico Nacional, Plan de San Luis y Díaz Mirón s/n, Col. Casco de Santo Tomas, Miguel Hidalgo, Mexico City 11340, Mexico; heisaac2013@hotmail.com (H.I.R.-G.); lidia_de_la_cruz@hotmail.com (L.E.D.l.C.-Á.); kammar_nutrition@hotmail.com (A.K.-G.); 2 Instituto Nacional de Geriatría, Anillo Periférico 2767, Col. San Jerónimo Lídice, La Magdalena Contreras, Mexico City 10200, Mexico; 3 Facultad de Química, Universidad Nacional Autónoma de México, Circuito Escolar S/N, Ciudad Universitaria, Coyoacán, Mexico City 04510, Mexico; cani@unam.mx; 4 Instituto Nacional de Medicina Genómica, Periferico Sur 4809, Col. Arenal Tepepan, Tlalpan, Mexico City 14610, Mexico; 5 Unidad de Investigación en Farmacología, Instituto Nacional de Enfermedades Respiratorias, Ismael Cosio Villegas, Secretaría de Salud, Calzada de Tlalpan 4502, Col. Belisario Domínguez Sección XVI, Tlalpan, Mexico City 14080, Mexico; pharman007@hotmail.com (J.C.H.-C.); linamarcelaba@yahoo.com.mx (L.M.B.-G.)

**Keywords:** interindividual variability, obesity, phentermine, tolerance, weight loss success

## Abstract

The efficacy of anti-obesity drugs usually does not consider the high degree of interindividual variability in responses to the drug which could affect the decision to withdraw the drug early due to ineffectiveness or to continue therapy according to specific expectations of success. The aim of this study was to analyze body weight loss in kilograms during the first month (1 mo-BWL_kg_) of treatment with 30 mg phentermine and development of tolerance to phentermine, on its 6-month efficacy. One hundred sixty-six subjects with obesity were individually or jointly analyzed in the study. Subjects with 1 mo-BWL_kg_ of <1 kg, 1–3 kg, 3–5 kg, and ≥5 kg reached 6-month mean percentage body weight reductions (BWR%) of approximately 3%, 5%, 10%, and 15%, respectively. Development of late tolerance (4–6 months) to phentermine had a lower impact than early tolerance (2–3 months). Subjects with 1 mo-BWL_kg_ < 3 kg who developed early tolerance did not achieve relevant BWR% (≥5%) at month 6, while the rest of the subgroups achieved increasing and progressive BWR%, according to their 1 mo-BWL_kg_ range and time of onset of tolerance. The 1 mo-BWL_kg_ and development of tolerance to phentermine could be useful to predict the expected 6-month efficacy trends in obese patients treated with 30 mg phentermine.

## 1. Introduction

Obesity is a complex, multifactorial, and highly prevalent disease that should be treated accordingly [1]. Initial standard treatment of obesity is based on diet, exercise, and behavior modifications; however, benefits are often marginal. Therefore, drug therapy is frequently used to treat this disease [2,3].

The efficacy of anti-obesity drugs has been generally evaluated and reported based on average weight reductions (kilograms), average percentage weight loss, or other anthropometric measures. Nonetheless, high interindividual variability limits the usefulness of these parameters [4].

Even when obesity treatment guidelines by expert committees consider weight losses between 5% and 10% to be sufficient to translate into health benefits [5], efficacy expectations of anti-obesity therapies may vary depending on the magnitude of the weight reduction being considered. The decision to initiate drug therapy for obesity could be guided by the intention to improve chronic diseases or reduce the risk of developing comorbidities such as diabetes mellitus, hypertension, hyperlipidemia, among others, which may be associated with the magnitude of percentage body weight loss [6]. In addition, preoperative weight loss is recommended before bariatric surgery and other procedures like orthopedic hip or knee replacement, for which certain levels of body weight reduction in a specific period of time are required [7].

Although the efficacy of various anti-obesity drugs has been related to specific predictors such as the phentermine/topiramate, for patients who report eating disorders [8], naltrexone/bupropion, for patients who struggle with addiction [9,10], or even a cutoff of 1.81 kg weight loss in the first month of treatment, for most obesity drugs, these have not been sufficiently studied. Better criteria for the early identification of potential responders and non-responders to anti-obesity drugs are required since they would allow clinicians to select an appropriate drug or decide whether to modify the initial therapy according to response to therapy, thereby preventing adverse events and patient disappointment due to treatment failure [11]. In this sense, the search for predictors of antiobesity efficacy has included the glucagon-like peptide-1 receptor (GLP-1) agonist liraglutide, for which a weight loss ≥ 4 kg after the first month of treatment has a good predictive value for achieving the ≥5% BWL efficacy endpoint [12].

Phentermine is a noradrenergic drug which acts on the sympathetic nervous system by causing an increase in norepinephrine release. The release of this neurotransmitter leads to appetite suppression and increased resting energy expenditure [11,13,14]. Phentermine is indicated for short-term use (12 weeks), alongside lifestyle changes, to improve weight loss in obese subjects; drug discontinuation is recommended if tolerance to phentermine is developed [7]. Moreover, it has been suggested that a first month body weight reduction of around 2 kg, predicts the subsequent efficacy of phentermine [11].

Body weight loss in kilograms during the first month (1 mo-BWL_kg_) and development of tolerance to phentermine are variables of which analysis could derive in a better understanding of efficacy trends of this drug in different classes of obese patients and could be used to inform treatment expectations.

The purpose of this study was to explore the impact of 1 mo-BWL_kg_ and development of tolerance to phentermine on the six-month efficacy tendencies and safety of phentermine in obese subjects treated with 30 mg phentermine, to provide clinicians with a picture of the pragmatic use of this old drug.

## 2. Materials and Methods

### 2.1. Study Design

In this prospective, phase IV open-label study, 166 volunteers aged up to 59 years and with a BMI ≥ 30 kg/m^2^ were recruited to evaluate the six-month efficacy and safety of oral administration of 30 mg phentermine in obese patients. Exclusion criteria were hypersensitivity to sympathomimetic drugs, patients receiving other anti-obesity drugs, and patients diagnosed with any lung, kidney, liver, endocrine, or cardiac disease, psychiatric disorders, history of substance abuse, pregnancy or subjects who had taken any anti-obesity medication within the last 3 months. Weight stability prior to enrollment was not considered. The baseline health status of patients was determined by medical history taking and clinical examination. Patients received medical support and were instructed to follow a diet of 1500 kilocalories and to perform physical activity for 20 min per day.

A capsule of 30 mg phentermine was administered orally every morning before breakfast. All capsules (Terfamex^®^, Mexico City, Mexico) were provided by Productos Medix, S.A. de C.V. Body weight and body mass index (BMI) were determined during monthly visits between months 0 to 6. Fasting plasmatic glucose (FPG), triglycerides (TG), total cholesterol, low-density lipoprotein-cholesterol (LDL-C) and high-density lipoprotein-cholesterol (HDL-C) were measured at 0, 3 and 6 months in the study. Safety was evaluated at every visit by directed anamnesis and physical examination, and by reviewing the patient’s diary.

At every visit, patients were required to empty their bladder upon arrival, dress with a hospital gown, and a nude body weight was obtained with a calibrated scale. Height was determined with the patient standing with the heels together, and the buttocks, shoulders, and head in contact with the stadiometer. Measurements of systolic blood pressure (SBP) and diastolic blood pressure (DBP) were obtained with an electronic sphygmomanometer. FPG, TG, total cholesterol, LDL-C, and HDL-C were determined by standard laboratory blood chemistry measurements.

The body weight loss in kilograms (BWL_kg_) was calculated by subtracting the weight at the beginning of the study from weight at any other given time (months) in the six-month study period. Percentage body weight reduction (BWR%) was calculated with the percentage of weight lost for every month with respect to baseline weight. Patients were first grouped according to their 1 mo-BWL_kg_ as follows: <1 kg, 1 to <2 kg, 2 to <3 kg, 3 to < 4kg, 4 to <5 kg, 5 to <6 kg, 6 to <7 kg, 7 to <8 kg, and ≥8 kg. These groups were further categorized into the following 4 ranges of 1 mo-BWL_kg_: <1 kg, 1 to <3 kg, 3 to <5 kg and ≥5 kg. The final efficacy of the treatment was evaluated with the percentage of patients who achieved a BWR% of ≥5%, ≥10%, or ≥15%.

Tolerance to 30 mg phentermine’s weight-reducing effect was defined as the absence of body weight loss for any given month with respect to the prior month. The patients were classified into 6 groups according to the month when they developed tolerance to phentermine or if tolerance did not occur. Early tolerance was considered when tolerance to weight-reducing effect occurred between the 2nd and 3rd months, whereas late tolerance was considered if it occurred between months 4 to 6.

### 2.2. Data Analysis

Descriptive data for quantitative variables are presented as mean and standard deviation (SD) or 95% confidence intervals (95% CI), and as frequencies with percentages for qualitative variables. Data from subjects with at least 80% treatment adherence were grouped by 1 mo-BWL_kg_, month of tolerance development, and obesity class. Several subgroups of subjects were formed for further analyses.

A Spearman correlation analysis was applied between BWL_kg_ and time of duration of treatment (months). The correlation between 6-month BWR% and 1 mo-BWL_kg_ was calculated with the Pearson’s correlation test. Data are presented as Spearman (ρ) or Pearson (r) correlation coefficients with their statistical significance values (*p*). Two linear regression analyses were applied to determine the effect of treatment time and 1 mo-BWL_kg_ on final BWL. The assumptions of the analysis were verified by residual analysis and collinearity tests; the validation of the model was performed through the determination coefficient. Data are presented as β coefficients, 95% CI, and *p* value.

Comparisons of quantitative variables between 3 or more groups were performed using fixed-effects ANOVA models with a Tukey post hoc test for pairwise comparisons; the comparisons between the 3-month and 6-month data were made using the paired *t*-test. Qualitative comparisons were made using the chi-square test or Fisher’s exact test.

Based on the impact of the 3 previously analyzed variables on the effectiveness of phentermine, 1 mo-BWL_kg_ groups (<1 kg, 1 to <3 kg, 3 to <5 kg and, ≥5 kg) were subdivided into 16 subgroups, according to the development of tolerance at month 2 (2 moT), month 3 (3 moT), late tolerance (LT), or no tolerance (NT). Only subjects with class 1 and class 2 obesity were included for these analyses. The BWR% was presented as mean with standard error (SE) and 95%CI, and as the percentage of subjects who achieved ≥5%, ≥10%, or ≥15% BWR%. Patients with class 3 obesity were evaluated similarly and separately.

Differences were considered statistically significant with a bilateral *p* < 0.05. Statistical analyses were carried out in the SPSS v. 22.0 software. Graphics were created in GraphPad Prism v.9.0.2.

## 3. Results

### 3.1. Demographic Data

Most patients were women (86.7%), aged between 30 and 49 years (66.2%), and had class 1 (52.7%) or class 2 (47.2%) obesity. Mean blood pressure, total cholesterol, LDL-C, and HDL-C were within normal reference values, although mean fasting glycaemia and triglycerides were slightly above reference values (Table 1).

### 3.2. Efficacy of Phentermine Treatment

**Figure 1:**
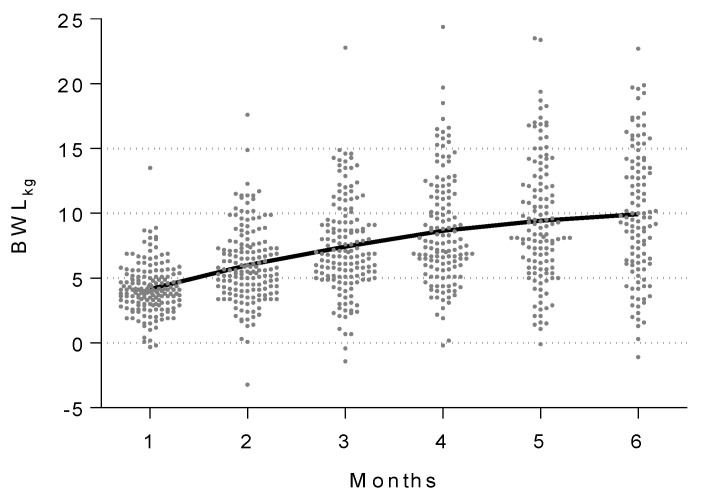
Variability in the body weight loss in kilograms (BWL_kg_) for every time point in the 6-month follow-up period, induced by 30 mg phentermine in 166 patients with obesity. Each point represents the weight loss by one subject for any given month, whereas the line shows the trend of average weight loss for every month. BWL_kg_ = Body weight loss in kilograms.

Phentermine treatment induced a BWL_kg_ of 7.5 (SD: 3.6) and 9.9 (SD: 5.0) kg, as well as a BWR% of 8.3 (SD: 3.8) and 11.2 (SD: 5.5) percent, after 3 and 6 months, respectively. The most important body weight-lowering effect of phentermine apparently occurred during the first three months, with a great interindividual variability (Figure 1). BWL_kg_ was positively correlated with treatment time (ρ = 0.5, *p* < 0.0001). The linear regression analysis showed that for each month of treatment a BWL_kg_ of 1.18 kg was achieved (β = 1.18, 95% CI: 1.03–1.33, *p* < 0.0001, R^2^ = 0.22).

### 3.3. Effect of 30 mg Phentermine on Cardiometabbolic Parameters

Phentermine therapy significantly improved anthropometric and cardiometabolic variables (waist circumference, fasting glycemia, tryglicerides, and total cholesterol) after 3 and 6 months of treatment, while diastolic blood pressure and HDL-cholesterol improved until the 6th month of therapy (Table 2). None of the clinical and demographic characteristics like age, gender or BMI were correlated with 1 mo-BWLkg nor the 6-month BWR% efficacy of phentermine.

### 3.4. Impact of 1 mo-BWL_kg_, Development of Tolerance, and Obesity Class on the Efficacy of Phentermine

**Figure 2:**
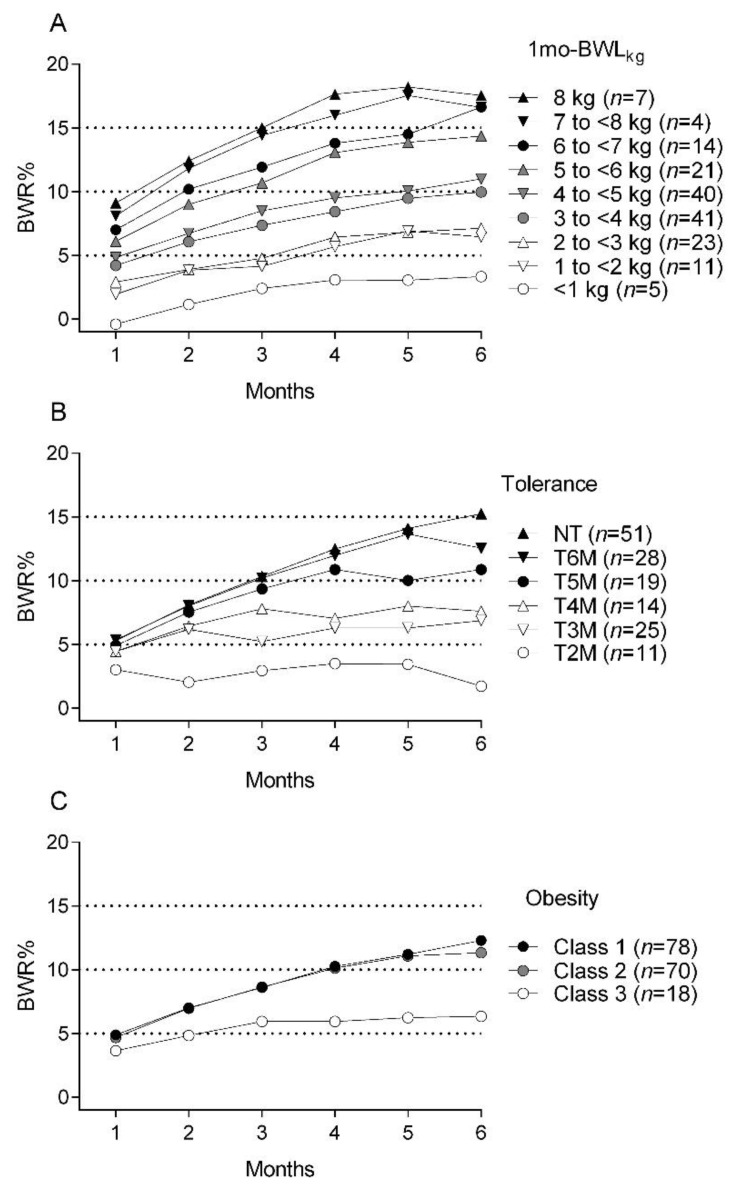
Six-month time course trends of 30 mg phentermine effect on percentage body weight reduction (BWR%) according to body weight loss in kilograms during the first month (**A**), development of tolerance to phentermine (**B**), and obesity class (**C**). Each point represents the mean. Standard deviations were omitted to allow visualization of overlapping traces. 1 mo-BWL_kg_: body weight loss in kilograms during the first month, kg: kilograms, *n*: sample size, NT: No tolerance, 6 moT: Tolerance at month 6, 5 moT: Tolerance at month 5, 4 moT: Tolerance at month 4, 3 moT: Tolerance at month 3, 2 moT: Tolerance at month 2.

The subclassification of patients according to 1 mo-BWL_kg_ exhibited a clear relationship with the subsequent efficacy of phentermine in terms of mean BWR% (Figure 2A). After grouping data into intervals according to the final BWR% efficacy measure, the mean BWR% at 6 months in subjects with a 1 mo-BWL_kg_ <1 kg was 3.3% (SD: 1.6); 1 to <3 kg, 6.9% (SD: 3.8); 3 to <5 kg, 10.0% (SD: 4.5); and ≥5 kg, 15.8% (SD: 4.2). The ANOVA model showed significant differences between all these groups (*p* < 0.0001), with the ≥5 kg 1 mo-BWL_kg_ group showing the highest 6-month BWR% (Tukey’s post hoc test: All *p* < 0.0001). Furthermore, 6-month BWR% had a positive correlation with 1 mo-BWL_kg_ (r = 0.6, *p* < 0.0001), with every 1 kg of 1 mo-BWL_kg_ causing a 6-month BWR% of 1.7% (β = 1.73, 95% CI: 1.33–2.14, *p* < 0.0001, R^2^ = 0.4).

Out of 148 subjects, 66% who completed at least 3 months of treatment developed tolerance to the weight-reducing effect of 30 mg phentermine at any given time point during follow-up: early tolerance (first 3 months) occurred in 25%, whereas late tolerance (4–6 months) was observed in 41%. A higher percentage of patients discontinued treatment after presenting early tolerance (47%) compared with those who developed late tolerance (13%). Patients who developed tolerance later than month 2 had a similar mean 6-month BWR% (Figure 2B).

Significant differences were among the different tolerance groups (*p* < 0.0001). The paired comparisons showed that subjects that did not develop tolerance had greater weight losses than those who did, except for 6 moT (NT: 15.3 ± 0.62%; 2 moT: 2.2 ± 0.9%, *p* < 0.0001; 3 moT: 7.1 ± 1.2%, *p* < 0.0001; 4 moT: 7.6 ± 0.8%, *p* < 0.0001; 5 moT: 10.9 ± 1.1%, *p* = 0.002 and; 6 moT: 12.5 ± 1.03%, *p* = 0.2). Nonetheless, there were no significant differences in mean BWR% for the immediate month before development of tolerance compared with 6-month BWR%.

Time course curves of BWR% according to obesity classes were similar for class 1 and class 2, but not for class 3 obesity. Mean BWR% for class 1 and class 2 obesity were >5% and >10% at 3 and 6 months, respectively. In contrast, patients with class 3 obesity had ~5% mean BWR% at both time points (Figure 2C). Through ANOVA modelling, patients with class 3 obesity had a lower BWR% than class 1 and 2 from months 2 to 6. (All *p* < 0.01); class 1 and class 2 obesity groups had similar BWR% for all months (All *p* > 0.05).

### 3.5. Three- and Six-Month Phentermine Efficacy Projections According to 1 mo-BWL_kg_, Development of Tolerance, and Obesity Class

**Figure 3:**
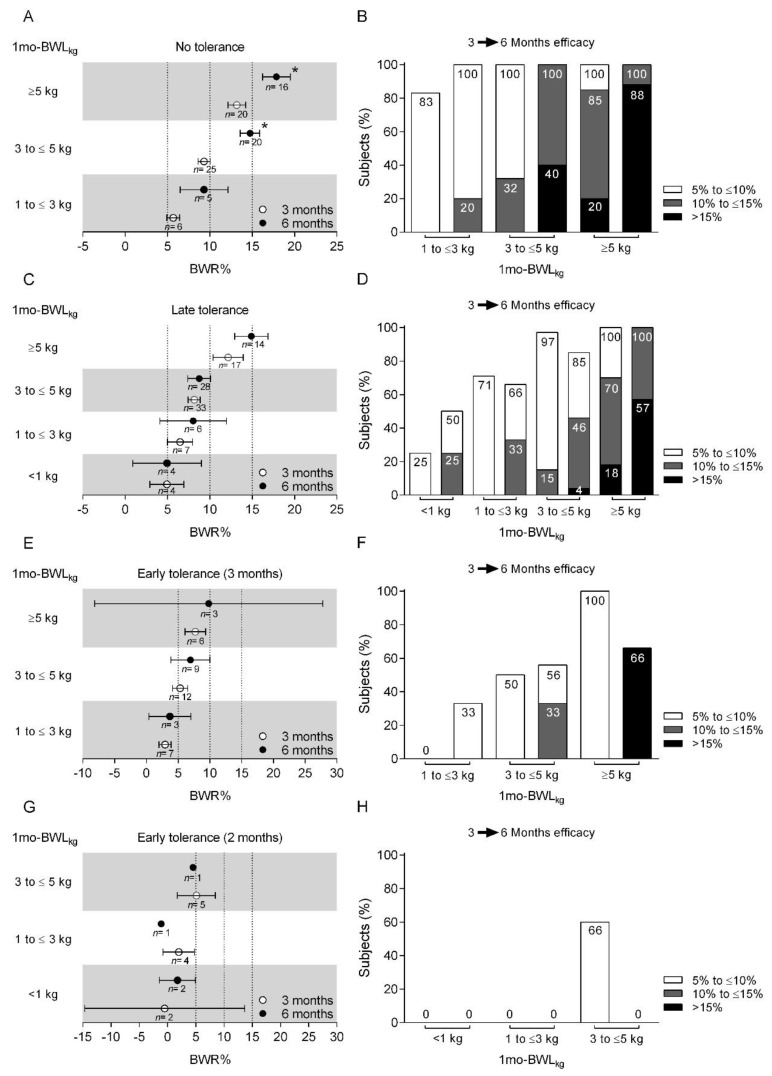
Three- and six-month mean percentage body weight reduction (BWR%) efficacy of 30 mg phentermine, according to body weight loss in kilograms during the first month (1 mo-BWL_kg_) and development of tolerance to phentermine. Tolerance was divided into no tolerance (**A**,**B**), late tolerance (**C**,**D**), tolerance at month 3 (**E**,**F**), and tolerance at month 2 (**G**,**H**). Each point represents the mean BWR% value and bars represent 95% confidence intervals (**A**,**C**,**E**,**G**) or the percentage of subjects who achieved a % interval of efficacy (**B**,**D**,**F**,**H**). * Significantly different by Student’s *t*-test (*p* < 0.05).

Subjects who did not develop tolerance to phentermine who had 1 mo-BWL_kg_ between the ranges 1 to <3 kg, 3 to <5 kg, and ≥5 kg showed proportional increases in 3-month mean BWR% (95% CI) of 5.6% (4.9–6.4%), 9.3% (8.6–10.0%) and 13.1% (12.1–14.2%), respectively, which increased by month 6 to 9.3% (6.4–12.1%), 14.7% (13.5–15.9%), and 17.8% (16.2–19.5%), respectively. Significant mean BWR% differences were found between months 3 and 6 for the 3 to <5 kg and ≥5 kg groups (*p* < 0.05) but not the 1 to <3 kg group. Almost 100% of subjects with 1 mo-BWL_kg_ of 1 to <3 kg achieved and maintained a BWR% of 5% to <10% between months 3 and 6, while those in the 3 to <5 kg and ≥5 groups significantly progressed from 5 to <10% and 10 to <15%, respectively, to 10 to <15% and ≥15% (*p* < 0.01). The NT group had no 1 mo-BWL_kg_ in the <1 kg range (Figure 3A).

Most patients with a 1 mo-BWL_kg_ of 1 to <3 kg and 3 to <5 kg had a BWR% efficacy of 5% to <10% after 3 months, of which 20% and 100%, respectively, achieved progress to the BWR% efficacy level of 10% to <15% after 6 months. Moreover, 40% of patients reached a BWR% efficacy level >15% in the 3 to <5 kg group at month 6. On the other hand, 85% of subjects with 1 mo-BWL_kg_ ≥5 kg achieved a BWR% efficacy level of 10 to <15% after 3 months, with significant progress towards >15% after 6 months (Figure 3B).

LT subjects with 1 mo-BWL_kg_ of <1 kg, 1 to <3 kg, and 3 to <5 kg reached proportional mean BWR% within the 5 to <10% range, with 3-month mean BWR% of 4.9% (2.9–6.9%), 6.4% (4.9–7.9%), and 8.1% (7.4–8.8%), respectively. Those with LT achieving a 1 mo-BWL_kg_ ≥5 kg had a 3-month mean BWR% of 12.1% (10.3–13.9%). In all groups, the same mean BWR% efficacy level was achieved, with discrete increases towards month 6: 4.9% (0.8–8.9%), 8.0% (4.0–11.0%), 8.7% (7.3–10.0%), and 14.8% (12.9–16.8%), respectively. There were no significant differences in the three-to-six-month comparisons (Figure 3C,D).

Patients in the ET group who developed tolerance at month 3 and achieved a 1 mo-BWL_kg_ of ≥5 kg were the only ones to reach a mean BWR% in the 5–10% range: 1 to <3 kg, 2.9% (2.0–3.9%); 3 to <5 kg, 3.7% (0.4–7.0%); and >5 kg, 7.7% (6.1–9.3%). Subjects maintained this BWR% efficacy up to the sixth month: 5.3% (4.1–6.5%), 6.9% (3.9–10.0%), and 9.8% (8.2–27.8%). There were no significant differences in three-to-six-month comparisons (Figure 3E). Approximately 50% and 100% of subjects with 1 mo-BWL_kg_ from 3 to <5 kg and ≥5 kg, respectively, achieved a 3-month mean BWR% in the 5 to <10% range. 

Only subjects with 2 moT who had a 1 mo-BWL_kg_ of 3 to <5 kg showed an efficacy level of 5 to <10 BWR% after 3 months of treatment, which represented 60% of patients in this group. However, they did not achieve the upper >10% range by month six (Figure 3G,H).

The comparison of all previous variables regarding BWR% in those who developed tolerance and those who did not showed only statistically significant differences for sex and mean baseline FPG, since 62.3% of women (81 out of 130) were tolerant against 88.8% of men (16 out of 18), at the six-month period of the study (*p* = 0.03), whereas mean baseline FPG was 107.6 mg/dL (SD: 3.1) in those who developed tolerance, compared with 98.9 mg/dL (SD: 1.9) in those who did not (*p* = 0.04).

### 3.6. Phentermine Efficacy in Class 3 Obese Subjects

Efficacy trends in subjects with class 3 obesity were obtained from a small sample. Only subjects with a 1 mo-BWL_kg_ ≥5 kg reached relevant BWR% (95% CI) efficacy levels: ET, 6.0% (1 subject) at month 3; LT, 9.8% (5.5–14.1%) at month 3 and 13.0% (12.6–13.4%) at month 6; and NT, 12.0% (18.7–42.6%) at month 3 and 19.6% (1 subject) at month 6. Significant differences were not found in 3-to-6-month efficacy comparisons. Notably, in this group was observed a mean FPG of 107.1 (SD: 23.1) mg/dL, and around 60% of subjects with FPG levels above 99 mg/dL. However, there were not found significant correlations between FPG baseline values and BWR% efficacy at 3 or 6 months. In addition, the only 2 no tolerant patients showed the best level of efficacy in the entire group, and one of them stayed in the study for only 3 months.

### 3.7. Safety of 30 mg Phentermine

A total of 803 adverse events (AEs) were reported for 166 patients receiving 30 mg phentermine during the 6-month follow-up period. Most AEs were mild, and only ~7.0% (mainly headache) were of moderate intensity. Table 3 shows the 15 most frequently reported AEs. No serious AEs were reported. AEs were mostly categorized as gastrointestinal, neurological, or psychiatric in origin. In addition, the ratio (95% CI) of total number of AEs/months of treatment with 30 mg phentermine for 2 moT, 3 moT, LT, and NT subjects were, respectively: 1.8 (0.4 to 3.1), 0.7 (−0.1 to 1.5), 0.8 (0.09 to 1.4), and 1.07 (0.25 to 1.9). There were no differences between subgroups.

## 4. Discussion

In the current study, we analyzed the individual and combined impact of the 1 mo-BWL_kg_ and development of tolerance to phentermine on the subsequent six-month efficacy and safety tendencies of 30 mg phentermine in obese subjects, seeking to expand criteria for the use of this drug according to BWL expectations. We selected the 30 mg phentermine dose in our study to increase the probability of having a suitable number of tolerant and non-tolerant subjects, since development of tolerance was our main variable of interest which has been reported to occur in a high proportion of patients treated with phentermine for three months at lower doses. Although phentermine is indicated for the short-term treatment of obesity, we sought to extend the study period to six months since longer treatment regimens are often used off-label in real-world clinical practice [15], thereby evidencing the need to obtain evidence from individualized data for its long-term safety and efficacy.

We found that daily oral administration of phentermine over a 6-month period led to weight reductions in a time-dependent manner, with a magnitude of 7.5 kg (8.3%) and 10 kg (11.2%), at months 3 and 6, respectively, although high interindividual variability was observed. Our data partially coincide with a study from Korea reporting a mean 8.1 kg weight loss after treatment with 30 mg phentermine for 12 weeks [16]. In a 6-month study of lower doses of phentermine, 7.5 mg and 15 mg phentermine led to a BWR% of 5.5% and 6.1%, respectively, compared with 1.7% for the placebo group [17]. High interindividual variability is a frequent phenomenon, not only in pharmacotherapy studies, but also in studies of lifestyle change interventions to lose weight [4,18]. In our study, 30 mg phentermine significantly improved parameters such as waist circumference, FPG, triglycerides, cholesterol, HDL-cholesterol, and diastolic blood pressure. A trend towards improvement in LDL-cholesterol and systolic blood pressure also occurred. Moreover, there were no cases of arterial hypertension reported. Although the evidence of 30 mg phentermine on other variables like blood pressure, lipid profile, or glycaemia is limited, our results are consistent with the improvement of glycemic parameters reported in previous studies, whereas the impact on blood pressure and lipids are inconsistent and smaller [19,20,21].

The current study provides evidence of good correlations between subjects according to their 1 mo-BWL_kg_ (<1 kg, 1 to <3 kg, 3 to <5 kg and, ≥5 kg) and BWR% efficacies of <5%, 5%, 10%, and 15% after 6 months of treatment with 30 mg phentermine. Similar results have been reported by Dhurandhar et al. [10] since 1 mo-BWL_kg_ showed a good correlation with BMI percentage reduction after six months of therapy with combined fenfluramine and phentermine. Currently, cut-off values of ~2 kg 1 mo-BWL_kg_ are used to define response to most anti-obesity drugs.

In this study, we also found that almost 66% of subjects developed tolerance to phentermine (25% and 41% at months 2 and 3, respectively), defined as a BWR% lower than or equal to that achieved in the previous month. Although this phenomenon has not been sufficiently studied, phentermine prescribing information mentions that tolerance to the anorectic effect may develop within few weeks after starting treatment which should make consider discontinuing the drug. Despite this, we also observed an acceptable level of phentermine efficacy in patients who developed late tolerance [22,23]. Thus, current prescribing information have probably only covered 12-week treatment with phentermine. Furthermore, we found that subjects who developed tolerance to phentermine reached a 6-month mean BWR% similar to that achieved the month before the presentation of this phenomenon, meaning that even if late tolerance is reached, high BWR% can still be achieved in a 6-month treatment course.

Patients with class 1 and class 2—but not class 3—obesity had similar BWL and time course effectiveness of phentermine. Although there are no reports of data on this regard for phentermine, it is generally accepted that greater effects of anti-obesity drugs can be achieved in subjects with class 3 obesity [11,24]. Due to the small number of subjects with class 3 obesity and high rate of voluntary withdrawals in our study, it is not possible to establish if 30 mg phentermine is less effective in patients with class 3 obesity. However, in the class 3 obesity group, the LT subjects or NT and 1 moBWLkg of ≥5 kg had a tendency towards increased efficacy around 10% and 15% of BWR% results at 3 and 6 months of treatment. Although it is tempting to suggest the apparent connection between the development of tolerance with higher blood glucose levels to explain the lower efficacy of phentermine 30 mg, not only in subjects with class 3 obesity, but in the general population studied, it should be considered that this last factor was also present in tolerant subjects who responded adequately to the drug. Thus, it is necessary to perform the analysis of a larger sample size to confirm if this could be mainly a class III effect.

Some guidelines for the treatment of obesity consider a 5–15% weight loss over a 6-month period to be of proven health benefit [25,26], yet maintenance of weight loss and prevention and treatment of comorbidities are considered the two main criteria for success. In addition, these guidelines recommend to assess pharmacotherapy efficacy after the first 3 months; if expected weight loss is achieved satisfactorily (>5% for non-diabetic and >3% for diabetic patients), treatment should be continued. Otherwise, treatment should be discontinued in non-responders [27,28,29,30].

The present study could help clinicians taking decisions according to BWL expectations of 6-month treatment with 30 mg phentermine, based on 1 mo-BWL_kg_ and development of tolerance. Early discontinuation of the drug in subjects with 1 mo-BWL_kg_ <1 kg and 1 to <3 kg who develop tolerance at months 2 and 3 could be reasonable, since they would not be expected to achieve a BWR% of at least 5%. Those with a 1 mo-BWL_kg_ of 3 to <5 kg or ≥5 kg who develop tolerance at months 2 and 3 show highly variable weight loss being lower than 10% after 6 months of treatment, and maintenance BWL compared with the prior month of onset of tolerance could be considered to decide continuing the drug according to BWL goals. This consideration could also be valid in subjects with class 3 obesity with acceptable 1 mo-BWL_kg_. Likewise, NT and LT subjects showed the best levels of proportional and progressive efficacy predictability between 5% and >15% after 3 and 6 months of treatment. Thus, clinicians could predict the potential efficacy of 30 mg phentermine according to the worst and best scenarios with their confidence intervals, alongside mean BWL_kg_ and BWR% efficacy.

Knowledge of the BWL trends of 30 mg phentermine, alongside an individualized evaluation of patients’ factors associated with weight loss (adherence to diet, physical activity, sex, age, metabolic conditions) would seem reasonable to take a final decision.

Although an important number of adverse events were reported during the 6-month treatment with phentermine, these were mostly mild in intensity, including dry mouth, hyperhidrosis, headache, dysgeusia, and constipation. The frequency of adverse events was not related to ET or LT. Even though no patients were withdrawn from the study due to adverse events, we could not determine if patients who withdrew voluntarily did it due to occurrence of AEs. The adverse event profile shown by 30 mg phentermine in this work was similar to those reported in other studies with high phentermine doses [19,20]. Interestingly, the disparity between the frequency of adverse events and development of tolerance suggests that the latter phenomenon may not be exclusively pharmacological in nature.

The main limitations of this prospective, phase IV open-label study include a small sample size, the lack of a control group, and that the recommended diet had the same energetic contribution for all patients. In addition, our study did not require weight stability prior to enrollment, which could bias the results; individuals who were already losing weight might develop tolerance to phentermine at a different rate than those with weight stability. Despite these limitations, we consider that this study provides a guide that could allow physicians to easily identify patients in whom phentermine will be effective in the long-term.

## 5. Conclusions

We confirmed that 30 mg phentermine is effective and safe during a 6-month treatment course and that weight loss in kg during the first month, alongside development of tolerance to phentermine at specific time could allow clinicians to predict drug efficacy.

## Figures and Tables

**Table 1:** Baseline characteristics of subjects in this study.

Parameter	Total Sample (*n* = 166)	Reference Value
Women, *n* (%)	144 (86.7)	-
Age, years (SD)	37.5 (9.7)	-
Weight, kg (SD)	86.2 (13.9)	-
Body Mass Index, kg/m^2^ (SD)	33.9 (3.7)	-
Class 1 obesity, *n* (%)	78 (47.1)	30–34.9 kg/m^2^
Class 2 obesity, *n* (%)	70 (42.)	35–39.9 kg/m^2^
Class 3 obesity, *n* (%)	18 (10.8)	≥ 40 kg/m^2^
SBP, mmHg (SD)	110.1 (11.0)	<120 mmHg
DBP, mmHg (SD)	76.7 (7.6)	<80 mmHg
Fasting glycemia, mg/dL (SD)	104.4 (25.4)	75–<99 mg/dL
Triglycerides, mg/dL (SD)	182.9 (113.0)	<150 mg/dL
Total Cholesterol, mg/dL (SD)	187 (38.4)	<200 mg/dL
LDL-Cholesterol, mg/dL (SD)	108.9 (34.8)	<130 mg/dL
HDL-Cholesterol, mg/dL (SD)	42.3 (9.5)	≥40 mg/dL

Data are expressed as mean (SD) or as frequencies (%). BMI: Body mass index; SBP: Systolic blood pressure; DBP: Diastolic blood pressure; LDL: Low-density lipoprotein cholesterol; HDL: High-density lipoprotein cholesterol; SD: Standard deviation.

**Table 2:** Anthropometric and cardiometabolic changes induced by 30 mg phentermine in subjects who completed at least 3 months of treatment.

Parameter	Baseline	3 Months	6 Months
Waist circumference, cm (SD)	110.6 ± 10.2	101.1 ± 10.0 *	97.8 ± 10.5 *
SBP, mmHg (SD)	109.9 ± 10.8	108.1 ± 8.9	107.3 ± 9.1
DPB, mmHg (SD)	77.0 ± 6.9	75.0 ± 7.7	74.6 ± 8.5 *
Fasting glycemia, mg/dL (SD)	104.7 ± 25.5	95.9 ± 16.7 *	91.5 ± 14.3 *
Triglycerides, mg/dL (SD)	184.9 ± 114.2	136.7 ± 123.5 *	137.8 ± 74.7 *
Total Cholesterol, mg/dL (SD)	188.0 ± 37.7	176.2 ± 38.7 *	173.3 ± 35.5 *
LDL-Cholesterol, mg/dL (SD)	108.7 ± 34.7	105.3 ± 30.3	99.9 ± 28.7
HDL-Cholesterol, mg/dL (SD)	42.4 ± 9.4	43.7 ± 9.7	45.8 ± 8.9 *

Data are presented as the mean (SD). SBP: Systolic blood pressure; DBP: Diastolic blood pressure; LDL: Low-density lipoprotein cholesterol; HDL: High-density lipoprotein cholesterol; SD: Standard deviation. * *p* < 0.05 by One-way ANOVA followed by Dunnett’s test.

**Table 3:** Main adverse events reported by patients with obesity who received 30 mg phentermine for 6 months.

Adverse Event	Total (%)	Mild (%)	Moderate (%)
Dry mouth	128 (15.9)	128 (15.9)	-
Headache	89 (11.1)	56 (7.0)	33 (4.1)
Hyperhidrosis	58 (7.2)	58 (7.2)	-
Dysgeusia	56 (7.0)	56 (7.0)	-
Constipation	47 (5.9)	41 (5.1)	6 (0.7)
Nervousness	41 (5.1)	41 (5.1)	-
Insomnia	36 (4.5)	36 (4.5)	-
Drowsiness	36 (4.5)	36 (4.5)	-
Thirst	35 (4.4)	35 (4.4)	-
Nausea	34 (4.2)	33 (4.1)	1 (0.1)
Anxiety	26 (3.2)	26 (3.2)	-
Fatigue	24 (3.0)	24 (3.0)	-
Irregular bowel sounds	15 (1.9)	14 (1.7)	1 (0.1)
Diarrhea	14 (1.7)	11 (1.4)	3 (0.4)
Abdominal pain	13 (1.6)	9 (1.1)	4 (0.5)
Others	151 (18.8)	142 (17.7)	9 (1.1)
Total	803 (100%)	746 (92.9%)	57 (7.1%)

Data are expressed as the number of adverse events, *n* (%).

## Data Availability

Raw data were generated at the National Institute of Respiratory Diseases, Ismael Cosio Villegas. Data supporting the findings of this study are available from the corresponding author on reasonable request.
